# Supplementary material for: Offspring BMI and lipid profiles following assisted reproductive technology: a comparative study of underweight and normal-weight mothers
Source: Lipids Health Dis. 2026 Jan 6;25:39. doi: 10.1186/s12944-025-02822-0 (PMC12874763; doi:10.1186/s12944-025-02822-0)
Supplement: Supplementary file 1 — Supplementary Material 1. [file 12944_2025_2822_MOESM1_ESM.pdf]

SPRINGER NATURE
Author Services

# Editing Certificate

This document certifies that the manuscript

## Offspring BMI and Lipid Profiles Following Assisted Reproductive Technology: A Comparative Study of Underweight and Normal-Weight Mothers

prepared by the authors

Zijing Wang, Wenxin Guo, Yujia Ren, Yiyuan Zhang, Jingmei Hu, Yue Liu, Linlin Cui

was edited for proper English language, grammar, punctuation, spelling, and overall style by one or more of the highly qualified English speaking editors at SNAS.

This certificate was issued on **November 20, 2025** and may be verified on the SNAS website using the verification code **378D-9CBC-9844-49FA-0832**.

Neither the research content nor the authors' intentions were altered in any way during the editing process. Documents receiving this certification should be English-ready for publication; however, the author has the ability to accept or reject our suggestions and changes. To verify the final SNAS edited version, please visit our verification page at secure.authorservices.springernature.com/certificate/verify.
If you have any questions or concerns about this edited document, please contact SNAS at support@as.springernature.com.

SNAS provides a range of editing, translation, and manuscript services for researchers and publishers around the world.
For more information about our company, services, and partner discounts, please visit authorservices.springernature.com.
