# Supplementary material for: Offspring BMI and lipid profiles following assisted reproductive technology: a comparative study of underweight and normal-weight mothers
Source: Lipids Health Dis. 2026 Jan 6;25:39. doi: 10.1186/s12944-025-02822-0 (PMC12874763; doi:10.1186/s12944-025-02822-0)
Supplement: Supplementary file 2 — Supplementary Material 2. [file 12944_2025_2822_MOESM2_ESM.docx]

**Supplementary Table 1 Associations of maternal BMI status and total offspring BMI and lipid profiles**

|  | **Maternal**  **Normal Weight**  **(n=3181)** | **Maternal Underweight**  **(n=315)** | **Crude Model 1 MD**  **(95% CI)^a^** | **Adjusted Model 2 MD**  **(95% CI)^b^** | **Adjusted Model 3 MD**  **(95% CI)^c^** | **Adjusted Model 4 MD**  **(95% C**I)**d** |
| --- | --- | --- | --- | --- | --- | --- |
| BMI z-score | 0.377 (1.24) | -0.267 (1.22) | -0.62(-0.78 -0.45)*** | -0.61(-0.77, -0.45)*** | -0.58(-0.75, -0.42)*** | -0.55(-0.71, -0.38)*** |
| TG, mmol/l | 0.729 (0.323) | 0.721 (0.311) | -0.01(-0.05, 0.03) | -0.01(-0.05, 0.03) | 0.00(-0.04, 0.04) | 0.00(-0.04, 0.05) |
| Total cholesterol, mmol/l | 4.04 (0.696) | 4.07 (0.647) | 0.01(-0.08, 0.10) | 0.01(-0.08, 0.10) | 0.03(-0.06, 0.12) | 0.04(-0.06, 0.13) |
| LDL-c, mmol/l | 2.35 (0.586) | 2.38 (0.581) | 0.01(-0.06, 0.09) | 0.01(-0.07, 0.08) | 0.03(-0.05, 0.11) | 0.04(-0.05, 0.12) |
| HDL-c, mmol/l | 1.47 (0.297) | 1.48 (0.280) | 0.007(-0.03, 0.05) | 0.01(-0.03, 0.05) | -0.00(-0.04, 0.04) | 0.00(-0.04, 0.04) |

Data are presented as mean (SD).

Mean differences were calculated by linear mixed models.

**^a^**Model 1: unadjusted.

**^b^**Model 2: adjusted for offspring age and offspring sex.

**^c^**Model 3: additionally adjusted for paternal age, maternal age, paternal BMI, parental education, maternal education, family incomes, paternal smoking, and paternal drinking.

**d**Model 4: fully adjusted model additionally adjusted for fertilization mode, stage of embryo transfer, embryo freezing, maternal hyperlipidaemia, gestational diabetes mellitus, medication for gestational diabetes mellitus, gestational age, delivery mode, parity, and offspring birth weight.

*Significant difference: *P* <0.05; **Significant difference: *P* <0.01; ***Significant difference: *P* <0.001

BMI, Body weight index; TG, Triglycerides; LDL-c, Low-density lipoprotein cholesterol; HDL-c, High-density lipoprotein cholesterol.

**Supplementary Table 2 Association of maternal BMI and its interactions with offspring BMI and lipid profiles**

|  | **Maternal BMI status *Offspring gender^a^** | **Maternal BMI status**  ***** **Fertilization mode^b^** | **Maternal BMI status**  ***** **Stage of embryo transfer^c^** | **Maternal BMI status**  ***** **Embryo freezingd** | **Maternal BMI status**  *** Paternal BMI^e^** |
| --- | --- | --- | --- | --- | --- |
| BMI z-score | 0.195 | 0.329 | 0.936 | 0.991 | 0.804 |
| TG | 0.382 | 0.340 | 0.261 | 0.253 | 0.911 |
| Total cholesterol | 0.026 | 0.896 | 0.705 | 0.685 | 0.311 |
| LDL-c | 0.021 | 0.731 | 0.727 | 0.689 | 0.506 |
| HDL-c | 0.580 | 0.396 | 0.412 | 0.525 | 0.211 |

Data are presented as interaction *P* value.

All interaction models were adjusted for the following common factors: offspring age, paternal age, maternal age, parental education, maternal education, paternal smoking, paternal drinking, family incomes, gestational diabetes mellitus, maternal hyperlipidaemia, medication for gestational diabetes mellitus, gestational age, delivery mode, parity, and offspring birth weight.

**^a^**Interaction Model 1: additionally adjusted for paternal BMI, fertilization mode, stage of embryo transfer, and embryo freezing.

**^b^**Interaction Model 2: additionally adjusted for paternal BMI, stage of embryo transfer, embryo freezing, and offspring sex.

**^c^**Interaction Model 3: additionally adjusted for paternal BMI, fertilization mode, embryo freezing, and offspring sex.

**d**Interaction Model 4: additionally adjusted for paternal BMI, fertilization mode, stage of embryo transfer, and offspring sex.

**^e^**Interaction Model 5: additionally adjusted for fertilization mode, stage of embryo transfer, embryo freezing, and offspring sex.

BMI, Body weight index; TG, Triglycerides; LDL-c, Low-density lipoprotein cholesterol; HDL-c, High-density lipoprotein cholesterol.

**Supplementary Table 3 Association of maternal BMI as a continuous variable with offspring BMI and lipid profiles**

|  | **Total offspring**  **β (95% CI)** | **Female offspring**  **β (95% CI)** | **Male offspring**  **β (95% CI)** |
| --- | --- | --- | --- |
| BMI z-score | 0.127(0.100 to 0.153)*** | 0.114(0.077 to 0.152)*** | 0.135(0.098 to 0.172)*** |
| TG, mmol/l | 0.003(-0.004 to 0.010) | 0.001(-0.008 to 0.010) | 0.004(-0.007 to 0.014) |
| Total cholesterol, mmol/l | -0.004(-0.020 to 0.011) | -0.024(-0.047 to -0.002)* | 0.010(-0.011 to 0.031) |
| LDL-c, mmol/l | -0.003(-0.016 to 0.010) | -0.020(-0.039 to -0.001)* | 0.009(-0.008 to 0.027) |
| HDL-c, mmol/l | -0.003(-0.010 to 0.004) | -0.007(-0.016 to 0.002) | 0.000(-0.009 to 0.009) |

β (95%CI) were calculated by linear mixed models.

Mean differences were calculated by linear mixed models.

All models were fully adjusted model adjusted for offspring age, offspring sex, paternal age, maternal age, paternal BMI, parental education, maternal education, family incomes, paternal smoking, paternal drinking, fertilization mode, stage of embryo transfer, embryo freezing, maternal hyperlipidaemia, gestational diabetes mellitus, medication for gestational diabetes mellitus, gestational age, delivery mode, parity, and offspring birth weight.

Model for total offspring additionally adjusted for offspring sex.

*Significant difference: *P* <0.05; **Significant difference: *P* <0.01; ***Significant difference: *P* <0.001

BMI, Body weight index; TG, Triglycerides; LDL-c, Low-density lipoprotein cholesterol; HDL-c, High-density lipoprotein cholesterol.
