# Supplementary material for: Offspring BMI and lipid profiles following assisted reproductive technology: a comparative study of underweight and normal-weight mothers
Source: Lipids Health Dis. 2026 Jan 6;25:39. doi: 10.1186/s12944-025-02822-0 (PMC12874763; doi:10.1186/s12944-025-02822-0)
Supplement: Supplementary file 3 — Supplementary Material 3. [file 12944_2025_2822_MOESM3_ESM.pdf]

Offspring BMI and Lipid Profiles Following Assisted Reproductive Technology: A Comparative Study of Underweight and Normal-Weight Mothers

By Yue Liu

WORD COUNT 4485
TIME SUBMITTED 26-NOV-2025 08:18PM
PAPER ID 119192095

Title: Offspring BMI and Lipid Profiles Following Assisted Reproductive Technology: A Comparative Study of Underweight and Normal-Weight Mothers

ABSTRACT

Background: The early developmental environment is associated with long-term metabolic health outcomes in children. A large proportion of underweight women tend to become pregnant via assisted reproductive technology (ART). However, the correlation between maternal prepregnancy underweight status and metabolic health in ART-conceived offspring remains poorly understood. The present study aimed to elucidate the associations of maternal underweight status with offspring development and lipid profiles, as well as to examine ART's interaction in this process.

Methods: The prospective cohort study included children conceived via ART at the Hospital for Reproductive Medicine Affiliated to Shandong University between April 2007 and August 2014. A total of 2,506 mother–offspring pairs, encompassing 3,496 visits, were analysed. Participants were divided into the following two groups based on the maternal body mass index (BMI): the normal weight group (18.5≤ maternal BMI <24 kg/m^2) and the underweight group (maternal BMI <18.5 kg/m^2). The BMI z-scores and serum lipid profiles of offspring aged 4–10 years were assessed.

Results: Compared with those of controls, the offspring of underweight mothers had significantly lower BMI z-scores (mean difference: -0.55; 95% confidence interval [CI]: -0.71 to -0.38). Interaction analysis revealed no significant interaction effect between ART treatment (fertilization mode, embryo transfer stage, or embryo freezing) and maternal BMI status on offspring BMI and lipid profiles. However, a significant interaction effect was observed between maternal BMI status and offspring sex on

offspring lipid profiles (interaction *P* values: 0.026 for total cholesterol and 0.021 for low-density lipoprotein cholesterol [LDL-c]). Compared with the control offspring, the female offspring of underweight mothers had higher total cholesterol levels (mean difference: 0.16; 95% CI: 0.02 to 0.29) and LDL-c levels (mean difference: 0.13; 95% CI: 0.02 to 0.25), while no such effects were found for male offspring (total cholesterol mean difference: -0.05; 95% CI: -0.17 to 0.09; LDL-c mean difference: -0.04; 95% CI: -0.15 to 0.07).

Conclusions: In the present study, there was an association between prepregnancy maternal underweight status and reduced BMI among ART-conceived offspring, and significant sex-specific differences in offspring lipid profiles were identified. Moreover, the analysis excluded any interaction effects between ART procedures and maternal BMI status on these outcomes. These findings emphasize the association of prepregnancy maternal underweight status with lower BMI and altered lipid profiles in ART-conceived offspring, providing crucial insights for preconception care and targeted postnatal prevention strategies.

Keywords: Underweight; Assisted reproductive technology; Mother-Child relationship; Lipids

Background

According to the developmental origins of health and disease (DoHAD) theory, environmental exposures in the early stages of life, including foetal period and early postnatal period, are crucial determinants of future health outcomes [1]. Extensive epidemiological studies have linked the preconception environment to the long-term health of offspring, with evidence indicating that preconception or early gestational body mass index (BMI), reflecting the nutrient environment of the mother, is essential for offspring development and metabolism [2]. Many studies have demonstrated that maternal obesity negatively affects foetal development, ultimately leading to adverse metabolic reprogramming [3, 4]. However, maternal underweight status and its possible links to offspring health have received limited attention. Underweight status is considered a potential risk factor for the "double burden of malnutrition" [5]. The prevalence of underweight status among adults is 6.8–7.7%), and remains a major challenge in low- and middle-income countries (LMICs) [6, 7]. Although low BMI is not equivalent to a clinical diagnosis of malnutrition, it serves as an initial proxy for assessing population-level nutritional challenges, especially in LMICs [8]. Data from South Asian countries show that more than 10% of women of reproductive age are underweight [9]. The prevalence of underweight among women is driven by various factors, such as economic backwardness, inadequate social protection, pandemic crisis, and cultural pressures, including unrealistic beauty standards perpetuated by the media [10, 11, 12, 13, 14]. Given these challenges, the observed associations between maternal underweight

status and offspring health warrant equally high levels of concern and further investigation.

Women who are underweight tend to experience reduced fertility, making it more challenging to conceive and increasing the risk of complications during pregnancy [15]. Among those who achieve natural pregnancy, prepregnancy underweight status is associated with an increased risk of preterm birth, small-for-gestational age (SGA), and low birth weight [16, 17, 18, 19]. Children born preterm or with low birth weight are associated with poorer cardiovascular health in adulthood, which is partly mediated by hypertension and adverse lipid metabolism originating in childhood [20, 21, 22, 23, 24, 25, 26]. However, limited research has specifically examined the long-term associations of maternal underweight status and offspring lipid profiles. Recently, with the increasing use of assisted reproductive technology (ART), a significant number of underweight women are now achieving pregnancy through ART [27, 28]. Since the first ART-conceived birth in 1978, ART has resulted in an estimated 8 million births worldwide from 1978 to 2014. In several countries (e.g., Denmark), ART-conceived infants accounted for >5% of annual births during this period [29]. More recent studies have indicated a rapid increase, with the cumulative number of ART births surpassing 10 million and potentially reaching 13 million in the past 40 years [30]. While ART has led to millions of births globally, the increasing use of this technology has raised concerns about the long-term outcomes of ART-conceived offspring [31]. Researches have

indicated altered cardiometabolic profiles in ART-conceived children [32], emphasizing the need for longitudinal safety assessments. Previous work has systematically investigated the adverse metabolic outcomes associated with maternal overweight and obesity in ART-conceived offspring, including adverse metabolic profiles in children conceived by frozen-transfer embryos [33]. However, the growth and lipid profiles of offspring born to ART-treated underweight mothers remain unclear, and potential interactions between ART procedures and maternal underweight status warrant investigation.

To investigate the childhood growth and lipid profiles in offspring of underweight mothers treated with ART, the present study was conducted using an ART birth cohort. The present study aimed to examine whether maternal underweight status is associated with offspring BMI and lipid profiles during childhood, as well as to explore potential sex differences and interactions with ART procedures. It was hypothesized that maternal underweight status before conception is associated with altered growth patterns and lipid profiles in ART-conceived offspring. Because existing studies rarely address the combined effects of maternal underweight status and ART, the present study focused on this specific population to explore their potential interaction on offspring lipid profiles.

Methods

Design and Study Population

The present study had a prospective cohort design. Patients who received ART at the

Center for Reproductive Medicine, Shandong University, and who had live births through April 2007 and August 2014 were prospectively recruited. ART treatment data and obstetric data were collected through the medical records system. In the present study, each child was invited for follow-up every three years between the ages of 4 and 10 years. However, the exact timing of visits was adjusted on the basis of the availability and comfort level of the children in consultation with their families. Repeated follow-ups during each period were encouraged, and the same procedure was utilized at the follow-ups. The study team contacted families via telephone and scheduled standardized clinical assessments at these time points in the hospital. During each visit, the children underwent anthropometric measurements and fasting blood draws conducted by trained nurses and laboratory technicians. Parents were also asked to complete updated questionnaires concerning sociodemographic and lifestyle factors. All the data were recorded in the ART birth cohort database. A total of 10,210 children aged 4–10 years were initially enrolled during the baseline phase. Twins and triplets (N=3,155) were excluded to minimize confounding effects associated with perinatal complications and growth restriction, which are more prevalent among multiples and can independently affect offspring metabolic outcomes [34]. Cases involving fertilization methods, such as artificial insemination by husband/donor, gamete intrauterine transfer, or oocyte donation (N=1,303), were excluded to ensure sample homogeneity and isolation of the effects of conventional ART procedures, namely, in vitro fertilization (IVF) and intracytoplasmic sperm injection (ICSI), as these methods involve different procedures and physiological

responses [35, 36]. Additionally, children with incomplete medical examination data (N=230) and unclear maternal physicians (N=106) were excluded because of incomplete baseline data, which could have compromised the accuracy and validity of the study results. Finally, mothers with a BMI of 24 kg/m^2 or higher (N=1,920) were excluded to focus specifically on the effects of maternal underweight status, reducing potential confounding from maternal overnutrition [33]. The process resulted in a final cohort of 2,506 mother–offspring pairs with 3,496 visits (Fig. 1).

Outcomes

At each visit, all participants underwent physical examinations for their anthropometric parameters and fasting blood tests for their lipid profiles. On the standardized scale, the height and weight of the children were measured repeatedly 26 while they were wearing light clothing and no shoes by trained staff. The average value was recorded. Each child's 11 body weight and height were recorded with a precision of 0.1 kg and 0.1 cm, respectively. BMI was derived as the following formula: body weight (kg)/height^2 (m^2). 1 Lipid profiles, including total cholesterol, triglycerides (TG), low-density lipoprotein cholesterol (LDL-c), and high-density lipoprotein cholesterol (HDL-c) levels, were assessed using homogeneous assays (Cobas c702 instrument; Roche Diagnostics) from fasting venous blood samples. Offspring BMI z-scores were calculated to eliminate the effects of age and sex using the following CDC formula on the basis of Chinese 17 L (Box-Cox transformation power), M (Median), and S (Generalized Coefficient of Variation)

reference data: BMI z-score = [((value/M)**L)-1]/LS [37, 38].

Exposures

Maternal BMI was calculated on the basis of prepregnancy anthropometric data. Similar to the measurement of BMI for children, maternal weight and height (accuracy ± 0.1 units) were assessed twice by experienced nurses during the initial clinical assessment, which occurred prior to the start of the ART procedures, and the recorded BMI provided a consistent measure of maternal anthropometric status prior to conception. Prior to measurement, participants were dressed in lightweight clothing and remained barefoot. Standard measuring equipment and uniform clinical procedures were employed to ensure data reliability. In the present study, maternal BMI was treated mainly as a categorical variable. The above-mentioned outcomes were compared between the two groups stratified by maternal BMI according to Chinese standards: maternal underweight (BMI <18.5 kg/m^2) and maternal normal weight ($18.5\leq$ BMI <24 kg/m^2). The maternal normal weight group served as the reference, while the underweight group was considered the exposed group.

Confounding Factors

literature reviews and analysis have suggested that several confounding factors are associated with maternal BMI and offspring lipid profiles. The BMI of the father was obtained from measurements taken at the same time as those taken for the mother. Data concerning parental age, parental education level, family income, maternal

hyperlipidaemia history, paternal smoking status, and paternal drinking status were collected by questionnaires before the initiation of ART. Data pertaining to gestational diabetes mellitus, medication use, fertilization modes, stages of embryo transfer, use of frozen embryos, gestational age, delivery mode, and parity were obtained from the ART and obstetric records. The sex and weight of children at birth were collected from birth records.

Statistics

For continuous data, results are shown as the mean (SD), and categorical variables are displayed as the frequency (percentage). In the linear mixed model and post hoc comparisons, the mean difference (MD) and 95% confidence interval (CI) between groups were calculated to assess the effects. Differences between groups were assessed through Student's t-test for continuous variables and the chi-square test for categorical ones. Linear mixed model 1 served as the basic model to assess differences in BMI and serum lipid levels between the offspring of underweight mothers and normal weight mothers. Linear mixed models 2–4 were used to progressively adjust for potential confounding factors, including offspring age, offspring sex, parental characteristics, socioeconomic status, ART procedures, obstetric history, and birth information. Detailed covariates for each model are listed in the footnotes of the tables. In addition to the results of the categorical analyses, maternal BMI was also treated as a continuous variable to explore its potential dose–response correlation with offspring BMI z-scores

and lipid profiles. Moreover, offspring sex, fertilization mode, stage of embryo transfer, embryo freezing, and paternal BMI were individually examined as interactive factors in interaction models. All interaction models were adjusted for offspring age, parental age, parental sociological factors, obstetric information and birth information, and each model was also adjusted for four other potential interactive factors. In all the models, the unexposed group with normal maternal weight was designated as the reference group. $P<0.05$ was considered to indicate statistical significance. R 4.3.0 statistical software was used for all the data analyses.

Results

Cohort characteristics based on maternal BMI status

Offspring were categorized into two groups on the basis of maternal BMI as follows: the maternal underweight group, which consisted of 209 offspring with 315 visits from underweight mothers; and the maternal normal weight group, which consisted of 2,297 offspring with 3,181 visits from normal weight mothers. Table 1 presents the population characteristics of the mother–offspring pairs in each group. Compared with those in the normal weight group, the maternal and paternal ages in the maternal underweight group were lower (28.9 vs. 30.1 for mothers, $P<0.01$; 29.7 vs. 31.0 for fathers, $P<0.01$). Although maternal BMI differed significantly between the groups (17.7 vs. 21.5, $P<0.01$), paternal BMI did not significantly differ ($P=0.26$). Additionally, there were no differences between the groups in terms of parental education level, family income, or

paternal history of smoking and drinking. The fertilization mode varied between the two groups; 65.8% of normal weight mothers and 56.5% of underweight mothers underwent in vitro fertilization (IVF),while 34.2% of normal-weight mothers and 43.5% of underweight mothers underwent intracytoplasmic sperm injection (ICSI) ($P<0.01$). The embryo transfer stage, embryo freezing rate, prevalence of gestational diabetes mellitus, prevalence of medication use, presence of maternal hyperlipidaemia, were similar in both groups The gestation week and mode of delivery did not significantly differ between the two groups. The proportion of firstborn children was greater among the offspring of underweight mothers (92.8%) than among those of normal weight mothers (87.9%, $P<0.01$). With respect to offspring characteristics, the birth weight of offspring of underweight mothers exhibited significantly lower values relative to those whose mothers had normal weight (3330 vs. 3410 g, $P<0.01$). There was no significant difference in sex at birth between the two groups, but the average age of the offspring in the underweight group was lower than that in the control group (5.33 vs. 5.56 years, $P=0.02$).

Offspring BMI and lipid profiles based on maternal BMI status

To investigate the development and lipid profiles of offspring of underweight mothers, the offspring BMI and blood lipid parameters were compared in the two groups, adjusting for potential confounding factors using linear mixed models (Supplementary Table 1). Offspring BMI was expressed as a z-score to account for the effects of sex and age on physical growth. Consistent with the maternal BMI, offspring of underweight mothers

had significantly lower BMI z-scores than offspring of normal weight mothers (MD: -0.62, 95% CI: -0.78 to -0.45). Furthermore, the association between maternal BMI and offspring BMI remained significantly positive after stepwise adjustments for offspring age, offspring sex, parental social determinants, family habits, ART and pregnancy factors. These findings indicated that the correlation between offspring BMI and maternal BMI remained robust even after considering multiple confounding factors. However, no differences were observed in offspring lipid levels, including blood triglycerides, total cholesterol, LDL-c and HDL-c levels, regardless of whether adjustments for confounding factors. These findings indicated that prepregnancy maternal underweight is associated with reduced BMI among ART-conceived offspring.

Associations of maternal BMI and its interactions with offspring BMI and lipid profiles

To further investigate the association between maternal BMI status and offspring health, interaction analysis was conducted to identify factors that interact with maternal BMI and influence offspring lipid profiles (Supplementary Table 2). The factors considered included offspring sex, ART procedures (fertilization mode, stage of embryo transfer, and embryo freezing), and paternal body mass index (BMI). None of these factors demonstrated interactions with maternal BMI on offspring BMI. Moreover, significant interactions were observed between offspring sex and maternal BMI concerning offspring lipid profiles, with an interaction *P* value of 0.026 for offspring total cholesterol and

0.021 for offspring LDL-c, suggesting that maternal underweight status may be associated with sex-specific differences in offspring cholesterol levels. However, ART treatment did not exhibit any interactive association with offspring lipid profiles, regardless of the fertilization mode (IVF vs. ICSI), stage of embryo transfer (cleavage vs. blastocyst), or embryo freezing status (frozen vs. fresh embryos). Additionally, paternal BMI did not interact with offspring lipid profiles. These findings indicated that maternal underweight status is associated with offspring lipid profiles through interactions with offspring sex, whereas ART treatment and paternal BMI do not interact with maternal BMI in this context.

Lipid profiles of offspring of different sexes

To further explore sex differences in offspring phenotypes, subgroup comparisons were conducted on the basis of sex, and several linear regression models were applied to adjust for multiple confounding factors by step (Table 2). Across all the models, the BMI z-scores were significantly lower for offspring of underweight mothers, regardless of sex and independent of adjustments for confounding factors. In the fully adjusted model 4, which accounted for offspring age, parental factors, parental social determinants, ART and pregnancy factors, the MDs of the BMI z-scores were -0.45 for female offspring (95% CI: -0.68 to -0.22) and -0.63 for male offspring (95% CI: -0.86 to -0.40). These results suggested that maternal underweight status is significantly associated with lower offspring BMI in both sexes, even after adjusting for potential confounders.

The lipid profiles differed between female and male offspring. Among female offspring, the mean values of total cholesterol and LDL-c levels tended to increase among the offspring of underweight mothers. After adjusting for offspring age, parental factors and parental social determinants, model 3 demonstrated significantly higher total cholesterol levels (MD: 0.14, 95% CI: 0.00 to 0.27) and LDL-c levels (MD: 0.12, 95% CI: 0.00 to 0.24) in offspring of underweight mothers than in those of controls. This effect persisted after additional adjustments for ART and pregnancy-related confounders. In model 4, the total cholesterol levels remained significantly higher in offspring of underweight mothers (MD: 0.16, 95% CI: 0.02 to 0.29), as did LDL-c levels (MD: 0.13, 95% CI: 0.02 to 0.25). However, no similar trends were observed in male offspring. When maternal BMI was analysed as a continuous variable, it was significantly positively associated with offspring BMI z-scores (β: 0.127, 95% CI: 0.100 to 0.153), male offspring BMI z-scores (β: 0.135, 95% CI: 0.098 to 0.172) and female offspring BMI z-scores (β: 0.114, 95% CI: 0.077 to 0.152). Conversely, maternal BMI was negatively associated with female offspring LDL-c levels (β: -0.024, 95% CI: -0.047 to -0.002) and female offspring total cholesterol levels (β: -0.020, 95% CI: -0.039 to -0.001), with no difference in male offspring (Supplementary Table 3).

All the models revealed that maternal underweight status was not significantly associated with lipid profiles in male offspring, regardless of adjustments for confounding factors. Together, these findings demonstrated that maternal underweight status is negatively associated with cholesterol levels, specifically in female offspring, and that parental age,

parental socioeconomic background, obstetric history, and birth information may serve as significant confounding factors in this correlation.

Discussion

The present study investigated the associations between ART-treated underweight women of childbearing age and offspring growth development and lipid profiles. Compared with control offspring, offspring born to underweight mothers presented significantly lower BMI z-scores, indicating increased susceptibility to growth stunting among offspring of underweight women [39]. Although the BMI z-scores among offspring of underweight mothers remained within the conventional "normal" range, the mean differences observed in both sexes could serve as early warning signs for potential nutritional or developmental concerns. Previous studies have shown that children with lower BMIs are more likely to experience delayed growth, impaired mental development, micronutrient deficiencies, impaired immune function, and increased infection risk [40, 41, 42, 43]. Therefore, even a modest reduction in the BMI z-score should not be overlooked in public health surveillance.

The lipid phenotypes differed, and sex was an interaction factor that modified the association between maternal underweight status and offspring cholesterol profiles. Female offspring of underweight mothers exhibited significant increases in total cholesterol and LDL-c levels, whereas no differences were observed in male offspring. Furthermore, interaction analysis revealed that ART procedures did not significantly

change the associations and suggested that certain factors, such as fertilization mode, embryo transfer stage, and embryo freezing, were not significantly associated with BMI or lipid profiles in the offspring of underweight mothers. These findings should be considered exploratory in nature but warrant confirmation in larger studies with stratified designs because of the limited population.

The association between maternal underweight status and offspring lipid profiles observed in the present study shares some similar patterns with findings from famine studies, providing historical insights into the developmental origins of metabolic risk. For example, in the Dutch Famine Birth Cohort, offspring whose mothers were exposed to famine prenatally and early in gestation had higher ratios of LDL-c to HDL-c than offspring of mothers who were not exposed to famine [44]. Although maternal underweight status is not equivalent to exposure to famine, such studies have pointed to the physiological circumstances associated with maternal underweight status before gestation that may be correlated to offspring lipid profiles, providing a conceptual framework for interpreting the present findings. The present results are further corroborated by animal studies indicating that maternal dietary alterations during pregnancy permanently affect offspring liver lipid metabolism [45]. In this context, researchers have proposed the “thrifty phenotype hypothesis”, suggesting that changes in offspring lipid profiles may be linked to the transition from maternal malnutrition early in pregnancy to improved nutrition postnatally [46]. Foetuses exposed to a malnourished

intrauterine environment adapt to these conditions and are at increased risk of adverse metabolic phenotypes, such as fat deposition, following postnatal catch-up growth [47]. Moreover, underweight women undergoing ART may increase their intake of high-energy foods during pregnancy to improve treatment efficacy and nutritional status [48], potentially influencing offspring cholesterol levels because of dietary changes. From an epigenetic perspective, genome-wide analyses of whole blood from the Dutch famine cohort have revealed an overall hypomethylated genome in females exposed to prenatal famine compared with controls, including differential methylation regions, such as *INSR* and *CPT1A*, which correlate with birth weight and serum LDL-c levels [49]. A potential link to reduced intake of methyl-sourced amino acids in famine-exposed mothers has been suggested [50].

Maternal obesity has been shown to have a sex-dependent association with offspring metabolism [51, 52], whereas little has been reported for mothers who are underweight. Another famine-related study has revealed that offspring of mothers with prenatal famine exposure exhibit long-term dyslipidaemia in adulthood, including elevated total cholesterol, triglycerides, and LDL-c levels, a pattern not observed in males or unexposed offspring [53]. This sex-specific variation in offspring lipids is consistent with the present findings. Given that the offspring in the present study were under 11 years old and that the mean values of LDL-c and total cholesterol in the offspring of underweight mothers were within normal physiological ranges, potential pathological changes in cholesterol

metabolism disorders in female offspring of underweight mothers after middle age warrant attention, which may lead to cardiovascular diseases, hyperuricaemia, and metabolic syndrome [54, 55]. These findings suggested that lipid monitoring and prevention of long-term chronic diseases in offspring of underweight mothers should be emphasized beginning in childhood. Inherent hormone-related differences in the expression and activity of proteins may be a source of sexual dimorphism [56]. A sex-stratified genome-wide association meta-analysis of lipid levels has revealed that approximately 3% to 5% of lipid-associated genetic loci exhibit significant sex-biased effects, with 21 novel loci newly identified on the X chromosome [57]. In addition, sex-specific placental regulation may contribute to these differences, as male and female placentas exhibit distinct gene expression profiles and metabolic responses to maternal metabolic status [58]. Maternal metabolic status also induces sex-specific epigenetic changes in the foetus, such as DNA methylation and histone modifications, which have long-term effects on lipid metabolism [59]. The above studies may account for the sex differences observed in the present study. Although most of these studies have emphasized sex-dependent differences in offspring outcomes in the context of maternal overweight or obesity, they nonetheless offer valuable insights and help guide future exploration of potential mechanisms underlying the present results.

Strengths and Limitations

The present study had several strengths. Given the increasing incidence of infertility and

the significant increase in offspring born via ART, ART offspring are inherently more susceptible to long-term cardiometabolic disorders compared with offspring from natural pregnancies [32, 60], and the associated risk factors in such a population need to be urgently evaluated. The present study was based on a large prospective ART birth cohort with more than seven years of follow-up and repeated measurements of BMI and lipid profiles, minimizing random error and enabling longitudinal assessment. A structured four-stage linear mixed model was applied to adjust for a wide range of potential confounders, with additional sex-stratified analyses to explore effect modification. These findings contribute to early risk identification and inform targeted preconception counselling by focusing on preconception underweight mothers. However, this study was limited by the small sample size of offspring born to underweight mothers, which may have reduced the ability to detect differences in lipid outcomes. While the study was sufficiently powered for anthropometric outcomes, the power for lipid profiles was limited. Nevertheless, the present cohort was established on the basis of one of the largest regional ART birth studies, and continued follow-up will help strengthen and validate these findings. Additionally, the present study relied on maternal/offspring BMI as the primary independent variable. While BMI is a standardized and widely used measure to facilitate direct comparison with similar approaches, future studies incorporating direct measures, such as body composition assessments and metabolic biomarkers, will be essential to more accurately characterize these associations. Furthermore, the absence of specific maternal nutritional data limited the ability to directly assess the specific

nutritional mechanisms linking underweight among gestational parents to offspring outcomes. In addition, given that there was no interaction effect between ART manipulation and maternal BMI on offspring lipid profiles in the present study, the phenotypes of offspring of underweight women who become naturally pregnant should be similarly depicted.

Conclusions

In conclusion, the present findings suggested that preconception underweight status among ART-treated mothers is associated with lower BMI in total offspring and higher cholesterol levels in female offspring, with no interaction effect from ART operations. This information may enhance the understanding of the health of ART offspring and provide direction for preconception care and prevention of potential diseases in the long term among offspring. Consequently, the present findings advocate the need for personalized weight surveillance in women at the pre-ART stage and early metabolic risk screening in ART-conceived children, particularly female offspring born to underweight mothers.

Offspring BMI and Lipid Profiles Following Assisted Reproductive Technology: A Comparative Study of Underweight and Normal-Weight Mothers

ORIGINALITY REPORT

12%
SIMILARITY INDEX

PRIMARY SOURCES

#	Source	Type	Words	%
1	www.ncbi.nlm.nih.gov	Internet	79 words	2%
2	www.researchgate.net	Internet	45 words	1%
3	www.science.gov	Internet	42 words	1%
4	"Abstracts of the 34rd Annual Meeting of the European Society of Human Reproduction and Embryology", Human Reproduction, 2018	Crossref	41 words	1%
5	pmc.ncbi.nlm.nih.gov	Internet	36 words	1%
6	www.frontiersin.org	Internet	28 words	1%
7	"Abstracts: Poster Presentations", Journal of Developmental Origins of Health and Disease, 2011	Crossref	25 words	1%
8	chs-nhlbi.org	Internet	24 words	1%

9	Geissler, Catherine, Powers, Hilary. "Human Nutrition", Human Nutrition, 2023<br>Publications	19 words — < 1%
10	"Diet, Nutrition, and Fetal Programming", Springer Science and Business Media LLC, 2017<br>Crossref	18 words — < 1%
11	www.researchsquare.com<br>Internet	17 words — < 1%
12	docksci.com<br>Internet	16 words — < 1%
13	www.mdpi.com<br>Internet	16 words — < 1%
14	G. Davey Smith. "Is there an intra-uterine influence on obesity? Evidence from parent-child associations in ALSPAC", Archives of Disease in Childhood, 6/26/2007<br>Crossref	14 words — < 1%
15	acervo.ufvjm.edu.br<br>Internet	14 words — < 1%
16	ovarianresearch.biomedcentral.com<br>Internet	14 words — < 1%
17	link.springer.com<br>Internet	13 words — < 1%
18	Austen D Le, Chiyuan Amy Zhang, Abby L Chen, Satvir Basran et al. "A Preconception Cohort Study of Nicotine Vaping and Incidence of Spontaneous Abortion", Nicotine and Tobacco Research, 2025<br>Crossref	12 words — < 1%

19	bmjopen.bmj.com<br>Internet	12 words — < 1%
20	www.gospel10.com<br>Internet	12 words — < 1%
21	Colleen Oliver, Helen Watson. "Omega-3 fatty acids for cystic fibrosis", Cochrane Database of Systematic Reviews, 2016<br>Crossref	11 words — < 1%
22	journals.sagepub.com<br>Internet	11 words — < 1%
23	www.sciencegate.app<br>Internet	10 words — < 1%
24	Bruno Basil, Jamila Aminu Mohammed, Izuchukwu Nnachi Mba, Ngozi Ijeoma Okoro et al. "Exploring the relationship between triglyceride-glucose-body mass index and hypertension in relation to cardiovascular disease risk in an African population of type 2 diabetes patients", Springer Science and Business Media LLC, 2025<br>Crossref Posted Content	9 words — < 1%
25	Yang Liu, Lu Ni, Chun Wang, Peng Shi, Liang Yin, Xiao-yan Fu, Hua-dong Zhou. "Association between internal carotid artery kinking and ischemic stroke: A population-based cross-sectional study", Journal of Stroke and Cerebrovascular Diseases, 2023<br>Crossref	9 words — < 1%
26	brieflands.com<br>Internet	9 words — < 1%
27	storage.googleapis.com<br>Internet	9 words — < 1%

28 Yunxi Li, In-Hee Lee, Sek Won Kong. "A sex-specific genome-wide association study of blood lipid levels in All of Us", Cold Spring Harbor Laboratory, 2025 — 8 words — < 1%
Crossref Posted Content

29 mhealth.jmir.org — 8 words — < 1%
Internet

30 worldwidescience.org — 8 words — < 1%
Internet

EXCLUDE QUOTES	OFF	EXCLUDE SOURCES	OFF
EXCLUDE BIBLIOGRAPHY	ON	EXCLUDE MATCHES	OFF
